# Evaluation of Code Blue Notifications and Their Results: A University Hospital Example

**DOI:** 10.5152/TJAR.2023.22965

**Published:** 2023-04-01

**Authors:** Songül Bişkin Çetin, Merve Gözde Sezgin, Mustafa Coşkun, Funda Sarı, Neval Boztuğ

**Affiliations:** 1 Department of Surgical Nursing, Akdeniz University, Faculty of Nursing, Antalya, Turkey; 2 Department of Internal Medicine Nursing, Akdeniz University, Faculty of Nursing, Antalya, Turkey; 3 ASAT General Directorate Information Technology Branch. Manager; 4 Department of Nephrology, Akdeniz University, Faculty of Medicine, Antalya, Turkey; 5 Department of Anaesthesiology, Akdeniz University, Faculty of Medicine, Antalya, Turkey

**Keywords:** Code blue, resuscitation, retrospective study, team

## Abstract

**Objective:**

Code blue is one of the important practices for preventing mortality and morbidity and increasing the quality of care in hospitals. The aim of this study was to evaluate the blue code notifications and their results, emphasise their importance, and determine the effectiveness and deficiencies of the application.

**Methods:**

In this study, all code blue notification forms recorded between January 1 and December 31, 2019, were examined retrospectively.

**Results:**

It was determined that code blue calls were made for 108 cases, including 61 females and 47 males, and the mean age of the patients was 56.47 ± 20.73. The accuracy rate of the code blue calls was determined as 42.6%, and 57.4% of them were made during non-working hours. Also, 15.2% of the correct code blue calls were made from dialysis and radiology units. The mean time for the teams to reach the scene was 2.83 ± 1.30 minutes, and the mean time to respond to correctly made code blue calls was 33.97 ± 17.95 minutes. It was found that 15.7% of the patients in correctly made code blue calls were exitus after the intervention.

**Conclusion:**

Early diagnosis of cardiac or respiratory arrest cases and quick and correct intervention are very important in achieving patient and employee safety. For this reason, it is necessary to continuously evaluate code blue practices, educate the staff, and organise improvement activities constantly.

**Main Points**

- Code blue practices are an important indicator of quality service in terms of patient and employee safety.
- In terms of patient and employee safety, it is important to evaluate the causes of code blue and the time to reach the scene, in terms of planning the improvement studies for the cause.
- It is necessary to share the shortcomings of code blue practices with the staff, to plan improvement activities constantly, and to make assessments regularly

## Introduction

Cases of cardiopulmonary arrest are common in hospitals.^1^ These cases account for 50% of all cardiovascular deaths and can cause approximately 230 000-350 000 deaths annually.^2^ In the event of arrest, one of the basic conditions for preventing mortality and morbidity is to respond to the case as soon as possible.^3,4^ Early recognition of cardiac arrest and correct and quick intervention are very important for bringing patients back to life.^5^ For this reason, the importance of code blue in hospitals is gradually increasing.^3,4^ Code blue is a warning system used in cases of cardiac or respiratory arrest that may be encountered in the hospital and has been created for giving an effective and quick intervention.^4^ It is the only code in the world where the same colour is used for the same emergency situation,^1^ and it aims to create a common language and establish fast communication between healthcare professionals.^6^ Considering the size of hospitals and the variety of services provided, it is important to educate health professionals to determine the code blue teams, establish the call system, and run the system appropriately.^4^

Code blue is considered an important quality criterion for hospitals.^7^ As a clinical quality indicator, it is recommended to analyse data regularly and to determine the causes of false calls.^6^ It is expected that the performance of the code blue system should be evaluated regularly,^8^ the problems occurring during its practice should be determined, and the system should be improved.^4^ It has been determined that there is a need for research to evaluate the effectiveness and applications of code blue teams hospitals.^1^ The correct use of the blue code system by health professionals, the status of teams to reach the case in the appropriate time and the evaluation of the time to intervene in the case are extremely important in terms of quality improvement activities. The aim of this study is to evaluate the blue code notifications and their results, emphasize their importance, and determine the effectiveness and deficiencies of the application.

## Methods

### Study Design and Setting

The study was conducted in a University Hospital that has 1149 beds and admits 1 168 952 patients annually on average. Because the hospital consists of different blocks and bed capacities and the number of patients admitted is high, 2 code blue teams work there. The code blue system was established in the hospital in December 2018. As of this date, the employees and code blue teams throughout the hospital were given education on how and in which situations the calls would be made. The correct blue code covers calls made in cardiac or respiratory arrest situations. All calls except in cases of cardiac or respiratory arrest are incorrectly identified as code blue. The code blue teams consist of at least 1 anaesthesiology and emergency physician, 1 paramedic, and 1 staff member, and they provide service 24/7. The teams respond to the code blue calls made from the blocks and garden areas determined for them. Telephone/radio is used for the code blue call system in the hospital. When a code blue notification is required, all healthcare professionals call 2222 (switchboard) from the nearest extension phone.

### Study Participants and Data Collection

In this retrospective study, all code blue notification forms recorded between January 1 and December 31, 2019, after the instalment of the code blue system were examined. The forms included the citizenship number and name and surname of the person responding to the case, the name and surname/file number of the case (if applicable), age of the case, the place where the call came from, the date and time of the call, the time of arriving at the scene, the intervention, the time when the intervention finished, and the result of the intervention. All the data collection forms covering all age groups filled out completely by the 2 code blue teams were included in the study. Intensive care units, emergency service units, and operating rooms perform cardiopulmonary resuscitation (CPR) themselves, and therefore, code blue teams are not called by these units. Thus, these units were not included in the study. The sample of the study consisted of 108 calls made using the code blue system.

### Ethics of the Study

Written approval of the Akdeniz Clinical Research Ethics Committee was obtained before starting the study (24.06.2020/428).

### Statistical Analysis

The Statistical Package for Social Sciences 23.0 software package (IBM SPSS Corp., Armonk, NY, USA) was used to evaluate the data obtained from the research. Conformity of continuous variables to normal distribution was examined by the Kolmogorov–Smirnov test. The categorical variables included in the study are presented as frequency (n) and percentage values (%), and continuous variables which met the parametric test assumptions are presented as mean ± SD values, while the variables that did not meet the assumptions are presented as median values (minimum–maximum). Pearson Chi-square and Fisher–Freeman–Halton exact test were used for the analysis of categorical variables. The Mann–Whitney *U*-test was used for the comparison of mean scores of 2 groups since parametric test assumptions were not met. The statistical significance level was accepted as .05 in the study.

## Results

In the study, code blue calls were made for 108 cases, including 61 females and 47 males. The mean age of the cases was 56.47 ± 20.73 years. The accuracy rate of the calls was 42.6%, and 42.6% of the calls were made during working hours. The teams were found to arrive at the scene in an average of 2.83 ± 1.30 minutes during code blue calls. It was determined that the average intervention time of the teams to correctly made code blue calls was 33.97 ± 17.95 minutes. As a result, 51.9% of the cases in code blue calls were taken to the emergency department, 15.7% were exitus, 25% were left in the scene, and 7.4% were taken to the intensive care unit (Table 1).

Correct code blue calls were mostly made from dialysis (15.2%) and radiology units (15.2%). The calls were most frequently made from the oncology clinic (8.7%). All units and the frequency of calls are shown in detail in Table 2.

Table 3 shows the distribution of code blue calls according to their causes. Accordingly, the rate of correct code blue calls due to cardiac/respiratory arrest was 42.6. The causes of calls that were evaluated to be incorrect and not a code blue call were worsening health condition, pain, syncope, dyspnoea, anxiety, seizures, palpitations, falls, and fatigue.

**Figure 1. f1-tjar-51-2-105:**
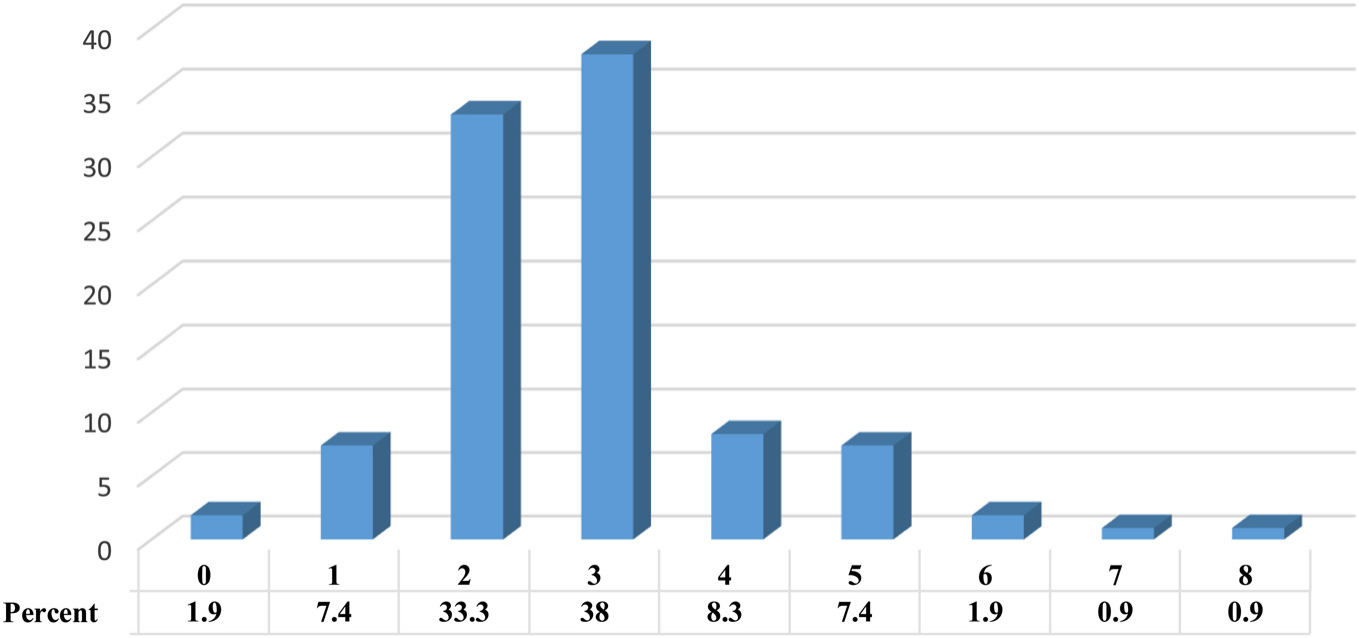
The time of arrival of the teams at the scene in code blue calls (minutes) (n = 108).

In Figure 1, the arrival times of the code blue teams at the scene are shown. The time of arriving at the scene in order of frequency was as follows: 3 minutes (38.0%); 2 minutes (33.3%); 4 minutes (8.3%); 1 minut‑e (7.4%); and 5 minutes (7.4%).

**Figure 2. f2-tjar-51-2-105:**
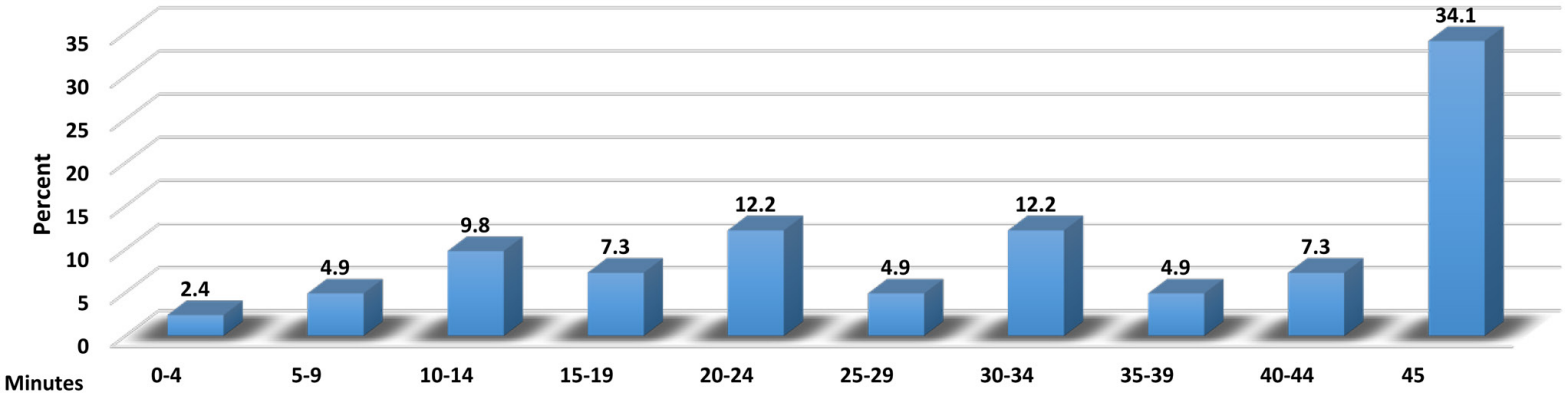
Time of responding to correctly made code blue calls (minutes) (n = 46).

In Figure 2, intervention times for correctly made code blue calls are shown. The time of intervention to the calls in order of frequency was as follows: 45 minutes or above (34.1%); 30-34 minutes and 20-24 minutes (12.2%); 10-14 minutes (9.8%); 15-19 minutes and 40-44 minutes (7.3%); 5-9 minutes, 25-29 minutes, and 35-39 minutes (4.9%); and 0-4 min (2.4%).

The characteristics of patients who were exitus are shown in Table 4. Of the total 46 correct code blue cases, 17 patients were exitus and 8 (47.1%) of them were females. The mean age of the patients who were exitus was 68.47 ± 19.94 years, and the youngest patient was 4 years old and the oldest 86 years old. The calls were mostly made from clinics (52.9%) and polyclinics (35.3). Of the 17 calls, 10 (58.8%) were made during working hours and 7 (41.2%) during non-working hours (Table 4).

## Discussion

Code blue application is an indispensable standard of hospitals,^9^ and the performance of the system should be evaluated periodically.^8^ In our study, the data of 108 code blue cases were analysed, and the mean age was found to be 56.47 ± 20.73 years[Table t1-tjar-51-2-105]. In the study of Özütürk et al.^10^ 225 code blue patients were evaluated in a hospital with 250 beds, and the mean age was found to be 54.1 years. Incesu et al^11^ found that the mean age of patients for whom a code blue call was made in a hospital with a bed capacity of 137 was 58.46 ± 20.81 years.^11^ Monangi et al^12^ stated that a total of 694 code blue calls were analysed in their study.^1^ In another study, it was reported that the number of patients for whom a code blue call was made was 237.^5^ In the study conducted by Topeli and Cakir,^8^ the data of 155 code blue patients were evaluated. When the result of our study is compared with the literature, it can be said that the number of code blue calls found in our study is relatively low and that the mean age is similar. We think that the sample size in our study was small possibly because the code blue system was newly established, the data were collected as of the date when the system was activated, and the awareness of the employees about the code blue application might not have been adequately developed.

It was found that 57.4% of the code blue calls in the data of our study were incorrect. In a study, 84.5% of the code blue calls were incorrect.^13^ Eroglu et al^1^ stated that 91.0% of the code blue activations in their study were incorrect.^1^ Çakırca and Kılcı^14^ reported the incorrect code blue rate as 67.1% in their study. When the literature is examined, it is seen that most code blue calls were made incorrectly, similar to our study. The communiqué on the code blue applications at the national level was published by the Ministry of Health in 2009.^15^ This shows that the code blue application process is new for hospitals. In addition, it is assumed in our study that the scope of the code blue application may not have been understood fully by the employees because the code blue system was established recently.

It was determined that 57.4% of the calls in our study were made during non-working hours[Table t1-tjar-51-2-105]. In a study, it was reported that 67.2% of code blue notifications were made during non-working hours.^10^ Esen et al^5^ stated that code blue calls were made most frequently between 10:00 pm and 12:00 am.^5^ Tosyalı and Numanoğlu^16^ reported that the rate of code blue calls made during non-working hours was 59%. In another study, it was stated that 56% of code blue calls were made during non-working hours.^17^ Çakırca and Kılcı^14^ reported that calls were frequently made during working hours, but the number of arrests was statistically higher during non-working hours. As a result of another study, it was determined that the code blue calls were received mostly between 6 am and 7 am.^18^ In our study, similar to the literature, it is seen that most of the calls were made during non-working hours. We think that this was because the number of healthcare professionals who can respond to the patient during non-working hours in the hospital where the study was conducted was less than that during working hours. This result reveals the importance of the need for the 24/7 operation of the code blue system.

In our study, it was determined that the mean time for the code blue teams to arrive at the scene was 2.83 ± 1.30 minutes [Table t1-tjar-51-2-105] and in 38.0% of the cases, it took the team 3 minutes to arrive at the scene[Fig f1-tjar-51-2-105]. When the literature is examined, it is seen that the times for code blue teams to arrive at the scene are 2.72 minutes,^17^ 2.02 ± 0.92 minutes,^18^ 1 minute 33 seconds, 1.10 minutes,^10^ and 40.77 ± 25.69 seconds,^17^ which are similar to the values in our study. The arrival of code blue teams at the scene within 3 minutes is considered a quality indicator.^19^ In our study, it is seen that the code blue teams reached the cases below the targeted time. This situation is extremely important in terms of patient and employee safety in the timely and rapid intervention to the arrest.

In our study, it was determined that the mean intervention time of the teams to the cases in the correct code blue calls was 33.97 ± 17.95 minutes [Table t1-tjar-51-2-105] and that the intervention time was 45 minutes or longer in 34.1% of the cases[Fig f2-tjar-51-2-105]. When the literature is examined, the mean intervention times of the code blue teams to the patients have been found as follows: 32.26 ± 13.47 minutes,^5^ 12.7 ± 12.7 minutes,^14^ 27.5 ± 11.4 minutes,^18^ 25.52 minutes,^20^ and 33.4 ± 19.6 minutes.^21^ The duration of CPR varies according to the condition of patients. Therefore, there may be differences in intervention times.

In our study, it was determined that correct code blue calls were mostly made from dialysis (15.2%) and radiology units and that 8.7% of them were made from oncology clinics[Table t2-tjar-51-2-105]. Özmete^22^ stated that 2.6% of code blue calls were made from the dialysis unit. In another study by İncesu,^11^ it was determined that code blue calls were frequently made from polyclinics. In other studies, it was found that the code blue calls were mostly made from the clinics,^5,21^ and these calls were mostly made from palliative^9,20^ internal medicine^16,20^ and oncology clinics.^20^ A review of the literature indicated that the frequency of code blue calls varied according to departments/units. In our study, especially the data of correctly made code blue calls were examined. We think that the reason why correct code blue calls were made from the dialysis, radiology, and oncology clinics was that the healthcare professionals working in these fields showed more interest in the education provided and that they were more aware of the code blue issue.

In our study, it was determined that 42.6% (correct calls) of code blue calls were made due to a cardiac arrest and that 27.8% (incorrect calls) of them were due to worsening health[Table t3-tjar-51-2-105]. In a study, it was reported that code blue calls were mostly made due to worsening health.^14^ In another study, it was reported that the most common causes of code blue calls were syncope (29.7%) and cardiopulmonary arrest (26.8%).^21^ In a study on the reasons for code blue calls, it was stated that 30 of the cases were cardiac arrest, 14 of them were convulsions, and 11 of them were syncope.^23^ In the study conducted by Topeli and Cakir,^8^ it was reported that 54.8% of the cases were exitus after the intervention. It is seen that our study result is similar to the literature.^8^

In our study, 51.9% of code blue cases were found to be taken to the emergency department. Similarly, 58.6% of code blue cases in a study were taken to the emergency department. In our study, 15.7% of the correct code blue cases ended with exitus, and 52.9% of the code blue calls for patients who ended with exitus were made from the clinics. In a study, it was determined that 57.2% of the cases in the code blue application showed no return of spontaneous circulation.^16^ In our study, most of the cases that were exitus in code blue calls were from the clinics, and we consider that this was because the patients hospitalized in the clinics were in a more critical condition. Rapid response systems used in hospitals are associated with lower rates of cardiopulmonary arrest and mortality in critically ill patients.^24^ For this reason, it is recommended to establish rapid response teams (rapid response system) to provide intervention before cardiac or respiratory arrest.

This study has some limitations. First, it is based on data obtained from the forms filled out by code blue teams. In addition, the code blue operation was newly established in the hospital where the study was conducted; therefore, this may have caused some shortcomings in practice. However, evaluation of the newly established system is also very valuable in terms of detecting problems and providing quick solutions.

## Conclusion

In conclusion, it was found in our study that code blue calls were mostly made for incorrect reasons. Since the code blue system was newly established and it was not fully understood, it is recommended to provide regular education for the personnel, increase practices that the personnel is involved in, and obtain opinions about code blue practices. It is necessary to share the shortcomings of code blue practices with the personnel, plan improvement activities constantly, and make assessments regularly. In addition, it is recommended to build a different team to respond to cases other than code blue. Code blue practices are an important indicator of quality service in terms of patient and employee safety.

## Figures and Tables

**Table 1. t1-tjar-51-2-105:** General Descriptive Information

Variable (n = 108)	n	%
**Gender**		
Female	61	56.5
Male	47	43.5
**Marital status**		
Single	47	43.5
Married	61	56.5
**Correctness of the calls**		
Correct	46	42.6
Incorrect	62	57.4
**Time of the call**		
Working hours (08:00 am to 04:59 pm)	46	42.6
Non-working hours (05:00 pm to 07:59 am)	62	57.4
**Referred units**		
Emergency service	56	51.9
Exitus	17	15.7
Undelivered	27	25.0
Intensive care unit	8	7.4
Variable (n = 108)	Mean ± SD	Median (Minimum-Maximum)
Age (years)	56.47 ± 20.73	59.5 (4-93)
Time of arriving at the scene (minutes)	2.83 ± 1.30	3 (0-8)
Time of responding for correct calls (n = 46) (minutes)	33.97 ± 17.95	34 (3-87)

**Table 2. t2-tjar-51-2-105:** Distribution of Correct Code Blue Calls by Units

Units (n = 46)	n	%
Dialysis unit	7	15.2
Radiology	7	15.2
Oncology clinic	4	8.7
Internal medicine clinic	3	6.5
Gastroenterology polyclinic	3	6.5
Chest diseases clinic	3	6.5
Neurology clinic	3	6.5
Children’s polyclinic	2	4.3
General surgery clinic	2	4.3
Inhospital areas^*^	2	4.3
Haematology clinic	2	4.3
Cardiology clinic	2	4.3
Orthopaedics clinic	2	4.3
Paediatric clinic	1	2.2
Eye clinic	1	2.2
Out-of-hospital areas^**^	1	2.2
Urology clinic	1	2.2

^*^Inhospital areas: blood bank, pharmacy, kitchen, genetics, forensic medicine morgue, laboratory, front of pathology, canteen, front of the intensive care unit, front of clinics, waiting rooms, and passages.

^**^Out-of-hospital areas: front entrance of blocks, garden, parking lot, canteen, and workshops.

**Table 3. t3-tjar-51-2-105:** Distribution of Code Blue Calls by Their Causes

Causes (n = 108)	n	%
Arrest	46	42.6
Worsening health condition	30	27.8
Pain	7	6.4
Syncope	7	6.4
Dyspnoea	5	4.6
Anxiety	4	3.8
Seizure	3	2.8
Dizziness	2	1.9
Palpitation	2	1.9
Fall	1	0.9
Weakness	1	0.9

**Table 4.** Characteristics of Exitus Patients in the Correct Code Blue Calls

Variable (n = 17)	n	%
Gender		
Female	8	47.1
Male	9	52.9
Unit where the call was made		
Clinic	9	52.9
Policlinic	6	35.3
Out-of-hospital areas—clinic	1	5.9
Inhospital areas	1	5.9
Time of the call		
Working hours (08:00 am to 16:59 pm)	10	58.8
Non-working hours (05:00 pm to 07:59 am)	7	41.2
Variable (n = 17)	Mean ± SD	Median (Minimum-Maximum)
Age	68.47 ± 19.94	72 (4-86)
